# Diagnosis of Arteriovenous Malformation in the Finger

**Published:** 2017-04-22

**Authors:** Ali Sawani, Katherine Huber, Sherma Zibadi, Wyatt G. Payne

**Affiliations:** ^a^C.W. Bill Young Bay Pines VA Medical Center, Bay Pines, Fla; ^b^Division of Plastic Surgery, Department of Surgery, University of South Florida Morsani College of Medicine, Tampa

**Keywords:** finger mass, arteriovenous malformations, AVM, vascular lesion, vascular abnormality

## DESCRIPTION

A 61-year-old right-handed man presented with a 2.5-cm subcutaneous mass to the dorsal radial aspect of the right long finger at the proximal interphalangeal joint. The painless mass had been present for 7 years without appreciable change in size. Pathology revealed an arteriovenous malformation (AVM) with no signs of malignancy.

## QUESTIONS

1. **What is an AVM and how is it classified?**
2. **What are common symptoms of AVM?**
3. **What imaging modalities can be used to diagnose AVM?**
4. **What are possible treatments of AVM?**

## DISCUSSION

AVMs can occur anywhere in the body but most often occur in the head and neck.^1^ Small AVMs may present as an incidental finding, whereas larger malformations may produce life-threatening complications such as high-output cardiac failure or impaired blood supply to a limb. AVMs are composed of small tortuous vessels comprising a nidus through which arteries, veins, and lymphatic vessels connect directly without a capillary bed.^2^ While AVMs in the digits are more frequently diagnosed as a congenital anomaly in children, acquired lesions may also be found in adult populations with a history of trauma or vascular procedures affecting the digit.

Patients with symptomatic AVMs most frequently complain of pain, decreased joint mobility, and soft-tissue or skeletal deformity and may have a history of cutaneous changes overlying the area. In asymptomatic individuals who present only with nodular subcutaneous mass, diagnosis is more difficult. The differential diagnosis includes giant cell tumor, glomus tumor, ganglion cyst arising from the joint or tendon sheath, and lipoma. Although often benign, AVMs can cause bone resorption or grow into adjacent tissue, leading to decreased blood flow, necrosis, and digit amputation.

Angiography is the current criterion standard, but it is often used to fully characterize the anatomy of the AVM after a diagnosis has been made. Radiographs are most often used as part of the initial workup of the nodular mass but are of limited utility and often nondiagnostic. Plain radiographs may demonstrate swelling around soft-tissue mass, phleboliths within the AVM, or osseous involvement demonstrated by areas of cortical thinning.^3^ Doppler ultrasound scan provides flow and velocity characteristics and is used primarily for diagnosis and postintervention monitoring. Contrast-enhanced computed tomography (CT) allows for accurate measurement of lesion size, as well as blood supply that feed and drain AVMs. CT scans can also be used to assess for acute hemorrhage or accompanying lesions that may not be visible on plain radiographs.^3^ Magnetic resonance imaging (MRI) offers numerous advantages over both CT and radiographs and has become the most important modality to diagnose AVMs.^4^ MRI techniques including time-of-flight and phase contrast can be used to isolate inflow blood supply to better predict risk of ischemia following embolization. MRI also provides well-defined images defining boundaries between the AVM, surrounding fat, and neurovascular bundles, which is necessary for planning of surgical or embolic interventions.^5^

Management of digital AVMs is largely dependent on the severity of symptoms. In asymptomatic patients, conservative management with surveillance is often sufficient. Patients who present with pain, ulceration, or signs of nerve compression may require intervention to save the digit and avoid amputation. Embolization or ethanol sclerotherapy may allow for closure of the AVM, but recurrence is common and repeat treatments are often required. In the digit, where AVMs are quite small, surgical resection may be a feasible option, as it removes all of the tissue involved in the AVM and has decreased recurrence.^6^

The low incidence of AVMs in the digit can present diagnostic and treatment challenges to clinicians. Treatment should be guided by the anatomy of the lesion and involvement of surrounding structures. Surgical resection is a curative therapy in symptomatic patients that maintains digit function and aesthetics.

## Figures and Tables

**Figure 1:**
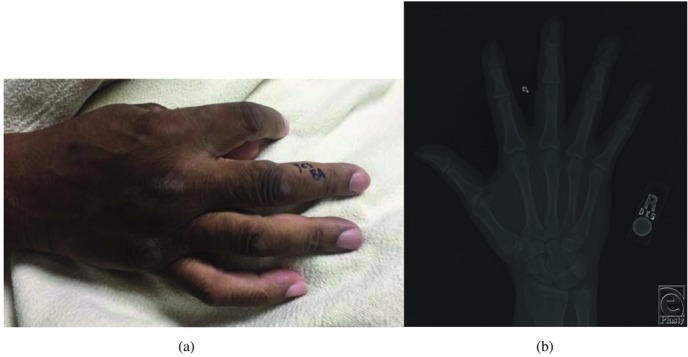
**(a, b)** Preoperative photograph and radiograph demonstrating a mass on the radial aspect of the right long finger. Radiograph demonstrates soft-tissue swelling, indicated by an arrow, without involvement of the proximal phalanx.

**Figure 2:**
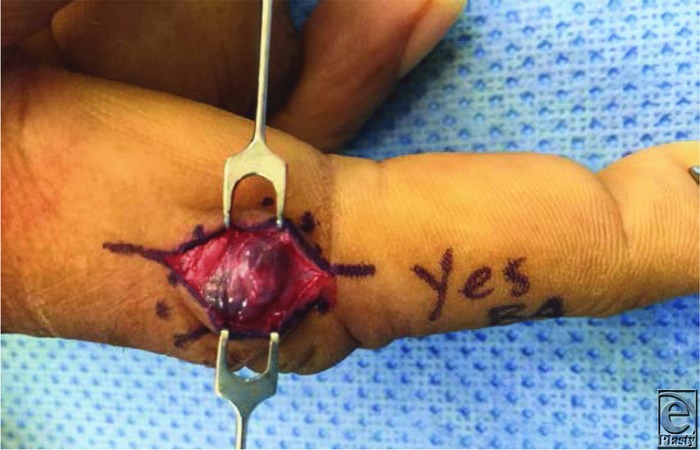
Intraoperative excision of the mass at the level of the proximal interphalangeal joint with preservation of the digital nerve.

**Figure 3:**
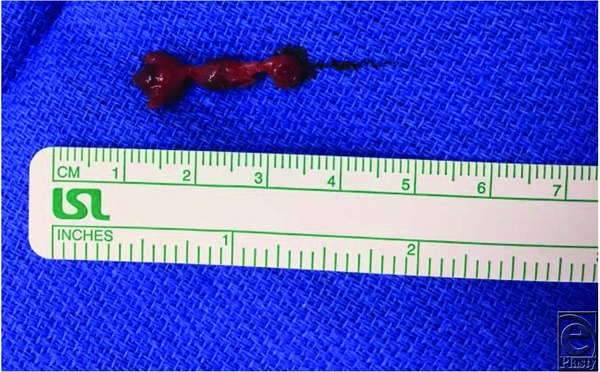
Gross image of the excised specimen showing 3 arteriovenous malformation distinct nidi.

**Figure 4:**
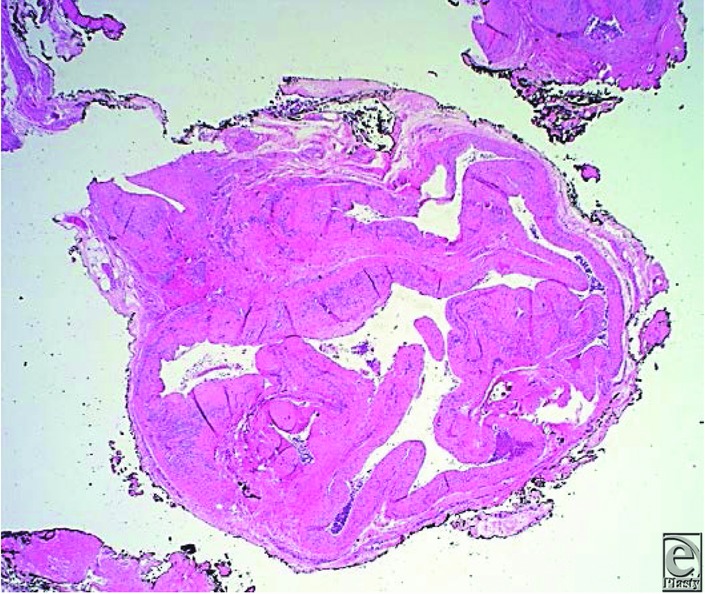
Pathology showing malformed blood vessels of intercommunicating arterial and venous structures with focal thrombus formation.

## References

[1] Dubois J, Soulez G, Oliva VL, Berthiaume MJ, Lapierre C, Therasse E (2001). Soft-tissue venous malformations in adult patients: imaging and therapeutic issues. Radiographics.

[2] Yakes WF (2004). Endovascular management of high-flow arteriovenous malformations. Semin Intervent Radiol.

[3] Hyodoh H, Hori M, Akiba H, Tamakawa M, Hyodoh K, Hareyama M (2005). Peripheral vascular malformations imaging, treatment approaches, and therapeutic issues. Radiographics.

[4] Legiehn GM, Heran MK (2010). A step-by-step practical approach to imaging diagnosis and interventional radiologic therapy in vascular malformations. Semin Intervent Radiol.

[5] Madani H, Farrant J, Chhaya N (2015). Peripheral limb vascular malformations: an update of appropriate imaging and treatment options of a challenging condition. Br J Radiol.

[6] Adani R, Busa R, Caroli A (1997). Microsurgical approach for a venous malformation of the thumb. J Hand Surg Br.

